# Synthesis and Properties of Magnetic Fe_3_O_4_/PCL Porous Biocomposite Scaffolds with Different Sizes and Quantities of Fe_3_O_4_ Particles

**DOI:** 10.3390/bioengineering9070278

**Published:** 2022-06-26

**Authors:** Jianhua Ge, Ramazan Asmatulu, Bo Zhu, Qiu Zhang, Shang-You Yang

**Affiliations:** 1 Department of Biological Sciences, Wichita State University, Wichita, KS 67260, USA; gejianhua@sdu.edu.cn; 2 Key Laboratory for Liquid-Solid Structural Evolution & Processing of Materials, Shandong University, Jinan 250100, China; 13605317708@139.com; 3 Department of Mechanical Engineering, Wichita State University, Wichita, KS 67260, USA; ramazan.asmatulu@wichita.edu; 4 School of Environmental Science and Engineering, Shandong University, Jinan 250100, China; zhangqiu@sdu.edu.cn; 5 Department of Orthopaedic Surgery, University of Kansas School of Medicine-Wichita, Wichita, KS 67214, USA

**Keywords:** Fe_3_O_4_ nanoparticles, PCL–HA scaffolds, magnetic scaffold, biocompatibility, cytotoxicity

## Abstract

In clinical practice, to treat diseases such as osteosarcoma or chondrosarcoma with broad surgical ostectomy, it would be ideal to have scaffolds that not only fill up the bone void but also possess the ability to regulate the subsequent regimes for targeted chemotherapy and/or bone regeneration. Magnetic targeting of therapeutic agents to specific sites in the body provides certain advantages such as minimal side-effects of anti-cancer drugs. The objective of this study was to characterize novel magnetic scaffolds that can be used as a central station to regulate the drug delivery of a magnetic nanoparticle system. Different sizes and quantities of Fe_3_O_4_ particles were mixed with poly-ε-caprolactone (PCL) to construct the magnetic scaffolds, and their mechanical properties, degradation performance, and cell biocompatibility were evaluated. It appeared that the presence of Fe_3_O_4_ particles influenced the magnetic, mechanical, and biological performances of the scaffolds. The prepared bio-nanocomposite scaffolds provided predominantly magnetic/superparamagnetic properties. Scaffolds with a micron-sized Fe_3_O_4_ to PCL weight (wt) ratio of 0.1:0.9 exhibited higher mechanical performances among samples, with Young’s modulus reaching 1 MPa and stiffness, 13 N/mm. Although an increased Fe_3_O_4_ particle proportion mildly influenced cell growth during the biocompatibility test, none of the Fe_3_O_4_/PCL scaffolds showed a cytotoxic effect.

## 1. Introduction

Development of appropriate scaffolds is a fundamental task in bone tissue engineering to help the body rebuild damaged or diseased tissues. During the bone healing process, a long regeneration time is expected to reestablish the complete functionality of damaged tissues. In many cases such as osteosarcoma or chondrosarcoma with broad excision treatment, it would be ideal to have scaffolds that possess the ability to regulate the delivery of localized, controllable, and long-term anti-cancer drugs and/or biochemical stimuli for bone regeneration [1,2,3,4].

Popular cancer drugs have been found to have applications in many locations of the body using various types of clinical medicine [5,6,7,8]. The best approach for treating tumors and other localized medical defects is to administer drugs only at the site of disease to reduce the side-effects of the cancer drugs. By delivering the drug locally, the toxicity of the drug to the rest of the body can be reduced while maintaining the desired therapeutic benefit at the site of interest. Many exciting drugs developed by the pharmaceutical industry have shown remarkable success during in vitro and in vivo testing but have yielded undesirable results in clinical trials due to systemic toxicity of the drug to other parts of the body. Thus, the ability to deliver large concentrations of drugs only at the site of treatment is of major importance for both the pharmaceutical industry and clinicians.

Magnetic targeting of therapeutic agents to specific sites in the body possesses certain advantages over other drug delivery methods [9,10,11]. One of them is that magnetic nanoparticles (MNPs) composed of magnetite are well tolerated by the human body [12,13]. Further, magnetic fields are well suited for biological applications and do not interfere with most biological processes [14,15]. Another advantage is that magnetic colloids can be injected into the bloodstream and guided to the targeted area with external magnetic fields [16,17,18,19]. However, there are many technical difficulties in many cases for patients to approach the external magnetic source and/or effectively receive the targeted drug delivery with the external magnetic field. The objective of the current study was to establish a poly-ε-caprolactone (PCL)-based bone scaffold with an internal magnetic capacity (distinct-sized magnetite particles—micron or nanometer ranges) to serve as a regulatory station to control the drug delivery efficiency of the magnetic nanoparticles.

Poly-ε-caprolactone (PCL) is one of the polyester polymers that has been approved by the FDA in clinical applications such as a cranial burr hole filler and trapezoid joint spacer. PCL has several advantages over other polymers including benign biocompatibility, low cost, biodegradability, and easy fabrication. Previous studies have suggested that PCL was a good candidate biomaterial for cartilage tissue engineering in terms of cell attachment, proliferation, and matrix production [20,21,22,23,24,25]. Favorable effects of PCL composites on osteoblasts were also demonstrated as a potential bone graft substitute [26,27,28,29,30]. Magnetite Fe_3_O_4_ nanoparticles are non-toxic to the human body and are found naturally in the environment [31]. They have been widely used in biotechnology, pharmacology, and biochemistry, including nucleic acid detection, cell separation, targeted drugs, immobilization, biosensors, water treatment, and magnetic resonance imaging contrast agent fields [32]. The current report assessed the Fe_3_O_4_/PCL magnetic scaffolds for their magnetic and mechanical properties, degradation performance, and their biocompatibilities.

## 2. Materials and Methods

### 2.1. Preparation of Fe_3_O_4_/PCL Scaffolds 

Fe_3_O_4_/PCL scaffolds were prepared using a salt leaching technique [30]. Briefly, Fe_3_O_4_ particles with distinct size ranges of <5 μm or <50 nm were mixed with PCL (Mn = 80,000, Sigma–Aldrich, St. Louis, MO, USA) in different weight to weight (wt/wt) ratios (Table 1). Sodium Chloride (NaCl) granules sized 355–500 μm were added to the Fe_3_O_4_/PCL mixture at 9-fold the total weight to generate a controlled porosity in the scaffold. PCL (10 g) was first dissolved in 60 mL of tetrahydrofuran followed by homogeneous mixing in Fe_3_O_4_ particles and NaCl granules until a viscous slurry developed. The mixture was cast into a mold to allow solvent evaporation in a fume hood, followed by rinsing in excessive distilled water to leach out the NaCl particles. Washout fluid was collected for the assessment of magnetic particles that leached out. The ultrastructure of scaffolds was observed using a scanning electron microscope (SEM). To ensure the proper ratio of Fe_3_O_4_ in the scaffolds after the leaching process, the following experiment was performed: the final product of scaffold samples in glass test tubes was treated with daily changes of fresh tetrahydrofuran (THF, Millipore Sigma–Aldrich, St. Louis, MO, USA) for 10 days to remove the organic polymer material. After evaporation of the solvent, the weight ratios of the test tubes before and after the 10-day THF treatment would be the actual magnetic particle concentrations in the PCL scaffolds.

### 2.2. Mechanical Properties of the Fe_3_O_4_/PCL Scaffolds

The mechanical properties of the scaffold samples (dimension 10 × 10 × 10 mm^3^) were estimated by an Electroforce 3200 Test System (BOSE, Eden Prairie, MN, USA). The mechanical data of the samples were collected when the height of the samples was compressed to 75% of the original height.

### 2.3. Magnetic Property of the Fe_3_O_4_/PCL Scaffolds

The magnetic property of the Fe_3_O_4_/PCL scaffolds was obtained from hysteresis loops recorded in an alternating gradient-force magnetometer (MicroMag AGM 2900, Lake Shore Cryotronics, Westerville, OH, USA) at room temperature.

### 2.4. Degradation Performance of the Fe_3_O_4_/PCL Scaffolds

The Fe_3_O_4_/PCL scaffolds (10 mm × 10 mm × 2.5 mm) were immersed in 5 mL of sterile distilled Dulbecco’s modified Eagle’s medium (DMEM) with 10% FBS plus streptomycin (100 mg/mL), and penicillin (100 U/mL) for up to 4 weeks. The scaffold samples were removed from the medium weekly for weight determination after rinsing with distilled water and were dried for 24 h at 37 °C. This process was repeated for 4 weeks, and the percentage of the samples’ degradation (D) was calculated by the following equation: D = (W_0_ − W*_S_*)/W_0_ × 100% (W*_0_* represents the initial sample weight while W*_S_* is the sample weight after the soaking periods of 1, 2, 3 or 4 weeks) [33]. The pH values of the immersion water with different samples were recorded weekly before the water was changed, using an AB15 pH meter (Fisher Scientific, Waltham, MA, USA).

### 2.5. Biocompatibility Assay of the Fe_3_O_4_/PCL Scaffolds

The biocompatibility of the scaffolds was evaluated by growing human fibroblast 3T3 cells (ATCC, Manassas, VA, USA) on the scaffold samples, as described previously [34]. Briefly, 10^5^ cells in 1 mL culture medium were put onto the testing scaffold material (10 mm × 10 mm × 2.5 mm) in each well of a 24-well plate, and the plate was incubated at 37 °C under 5% CO_2_ for 4 days. Cells without scaffold were used as controls. On the fifth day, 100 μL MTT (5 mg/mL) was added to each well for 6 h before being replaced with 1 mL of 10% sodium dodecyl sulfate (SDS) solution. The plate was incubated at 37 °C overnight. Then, 200 μL of the supernatant from each well was transferred into a 96-well plate the next day, and absorbance was read at 590 nm on a microplate spectrophotometer (Molecular Devices, San Jose, CA, USA, SPECTRA max, PLUS).

### 2.6. Cytotoxicity Assay of the Fe_3_O_4_/PCL Scaffolds

Each sample (10 mm × 10 mm × 2.5 mm) was immersed in 1 mL culture medium in a sterile tube for 24 h at 37 °C before the samples were transferred to clean tubes with the same amount (1 mL) of fresh medium. The same procedure was repeated daily, and the media left were collected as Day 1 to Day 7 release media. The 3T3 cells were seeded in a 96-well plate at 10^4^/100 μL medium/well for 24 h in a tissue culture incubator (37 °C and 5% CO_2_ in air) before replacement of the sample release media (100 μL medium/well). Controls were cells in fresh medium. Cells were then incubated at 37 °C and 5% CO_2_ for 2 more days, followed by the addition of 20 μL MTT (5 mg/mL) to each well. The 10% SDS treatment and optical density measurement at 590 nm were carried out as described above for the biocompatibility assay. The cytotoxicity index of the samples was calculated based on the MTT assay values, normalized by the control cell proliferation data. A threshold value of 0.7 was used to eliminate the noise interference.

### 2.7. Statistical Analysis

Three scaffold samples per group were tested for mechanical, magnetic, and biocompatibility properties, and 3 independent experiments per test were repeated for reproducibility purposes. Data were expressed as means ± standard deviations (SD). Statistical analysis was performed using SPSS version 23 (IBM, Chicago, IL, USA). Data comparison between µm and nm magnetite particles at an identical concentration was analyzed by Student’s t-test, while the comparisons among different concentrations of Fe_3_O_4_ were done using one-way ANOVA with LSD post hoc multiple comparison; *p* < 0.05 was considered statistically significant.

## 3. Results

### 3.1. SEM Analysis

**Figure 1:**
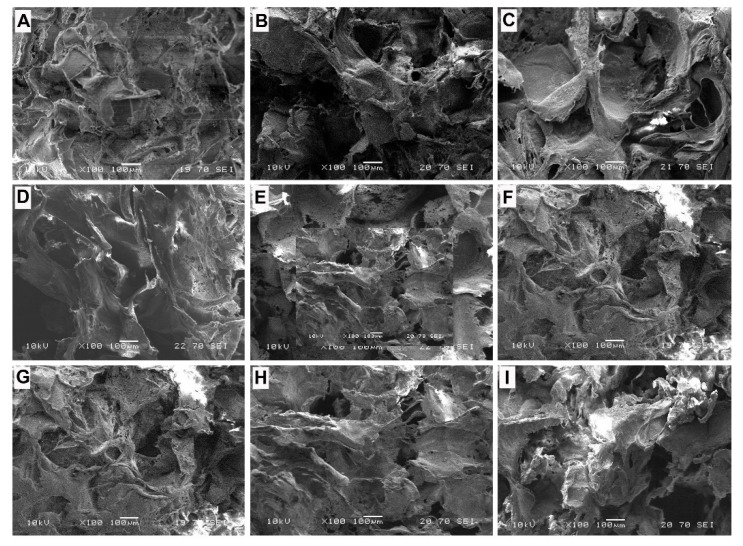
SEM images of the prepared samples: (**A**–**E**), scaffolds with micron-sized Fe_3_O_4_ particles (µm-1 to µm-5, sequentially); and (**F**–**I**), scaffolds with nanometer-sized Fe_3_O_4_ particles (nm-1 to nm-4).

The SEM pictures (Figure 1) indicate transfixion pores (size from 150 μm to 500 μm) in the scaffolds. At the same time, there were small pores (size from 5 μm to 100 μm) on the wall of large pores, which were produced by the evaporation of tetrahydrofuran. The high porosity configuration can help the growth of cells and tissue for scaffolding applications.

### 3.2. Mechanical Properties of the Scaffolds

**Figure 2:**
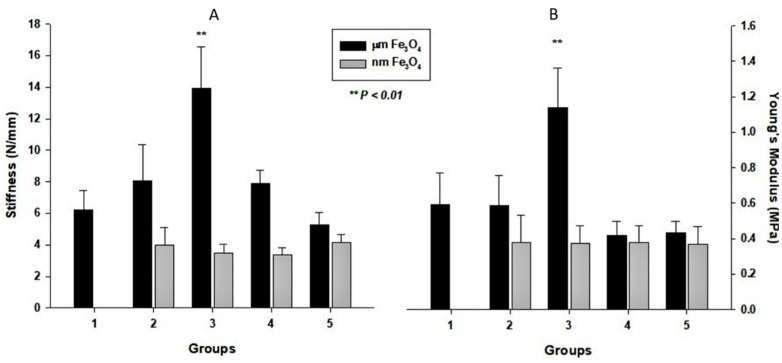
Mechanical properties of the prepared scaffold samples: Panel (**A**) exhibits the stiffness results of the samples; panel (**B**) shows the Young’s modulus results of the samples. Black column #1 is the pure PCL scaffold, black columns #2–#5 are samples with micron-sized Fe_3_O_4_ (μm-2 to µm-5 in sequence), and white columns are samples with nano-sized Fe_3_O_4_ (nm-2 to nm-5).

Figure 2 summarizes the mechanical performances of the prepared scaffolds. It was obvious that sample μm-3 had the highest mechanical performance among these samples. The series of nanoparticle Fe_3_O_4_ samples showed a lower mechanical performance compared to the series of micron-size Fe_3_O_4_ samples. It is worth mentioning that each component influences the mechanical performances of a bulk compound material. In the case of bulk compound materials, nano-inorganic powder can often work better than larger-sized inorganic powder to strengthen the mechanical performance of polymer materials. However, in our experiment, the results were contrary. The reason is that our samples had a high porosity, and the presence of the Fe_3_O_4_ particles wrecked the integrity of the pores’ walls of the PCL scaffolds to decrease the modulus of the PCL scaffolds. Further, difficulties in the dispersion of nanoparticles into the PCL scaffolds, agglomeration, and specific bonding may cause the reduction in the mechanical strength of the PCL scaffolds with nano-sized particles. However, because the hardness of the micron-sized Fe_3_O_4_ particles is higher than that of PCL, the micron-sized Fe_3_O_4_ particles can reinforce the PCL scaffold better. Thus, the mechanical performance of the scaffolds with micron-sized Fe_3_O_4_ particles can reach the vertex at the appropriate proportion of Fe_3_O_4_ particles (Fe_3_O_4_ particles: PCL wt/wt ratio of 0.10:0.90).

### 3.3. Magnetic Properties of the Scaffolds

**Figure 3:**
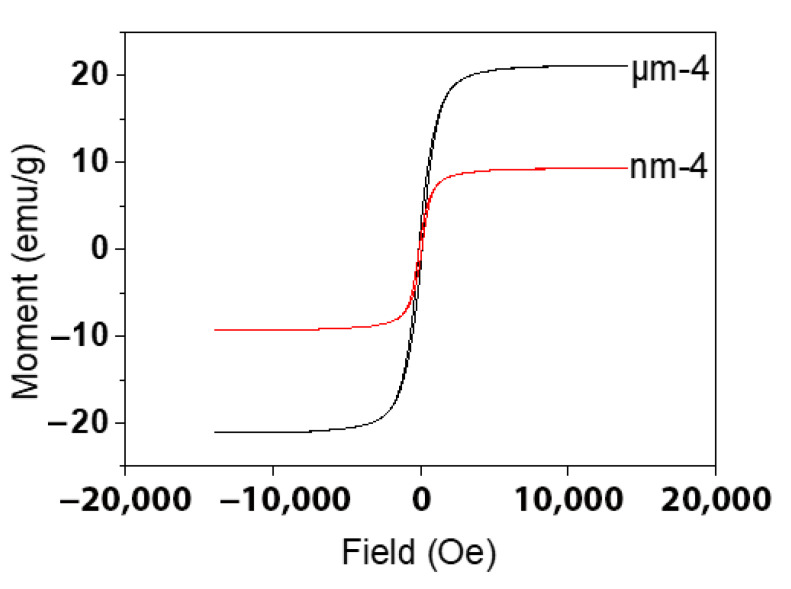
Hysteresis loops of the microcomposite scaffolds of the μm-4 and nm-4 samples.

The magnetic property of the Fe_3_O_4_/PCL scaffolds was characterized by magnetic hysteresis loops with varying magnetic fields at room temperature. Figure 3 plots the magnetization curves of the microcomposite scaffolds for sample μm-4 and sample nm-4. The saturation magnetization (Ms) of sample μm-4 was 21.09 emu/g, whereas the remanence magnetization (Mr) and coercive force (Fc) were 2.17 emu/g and 114Oe, respectively. However, the Ms of nanocomposite sample nm-4 was 9.28 emu/g while the Mr and Fc values were 1.18 emu/g and 98Oe, respectively. It is apparent that the magnetic performance of sample μm-4 was better than that of sample nm-4.

Compared with the Ms of bulk Fe_3_O_4_ (92 emu/g), the Ms values of the μm-4 and nm-4 samples were considerably lower, which might be attributed to the decreased particle size and the concomitant increase in surface area. The micro-sized magnetic samples behaved differently compared with the same concentrated samples with nanoparticle incorporation, which may be related to the magnetic to superparamagnetic transition and magnetic domain ordinations. It is known that the energy of a magnetic particle in an external field is proportional to its size due to the number of magnetic molecules in a single magnetic domain [35,36]. This can also explain the fact that the magnetic performance of the microcomposite sample μm-4 was higher than that of the nanocomposite sample nm-4 in our experiment, which may be because of the superparamagnetic effects (or size effects).

### 3.4. Magnetic Particle Retention and Degradation Performance of the Fe_3_O_4_/PCL Scaffolds

**Figure 4:**
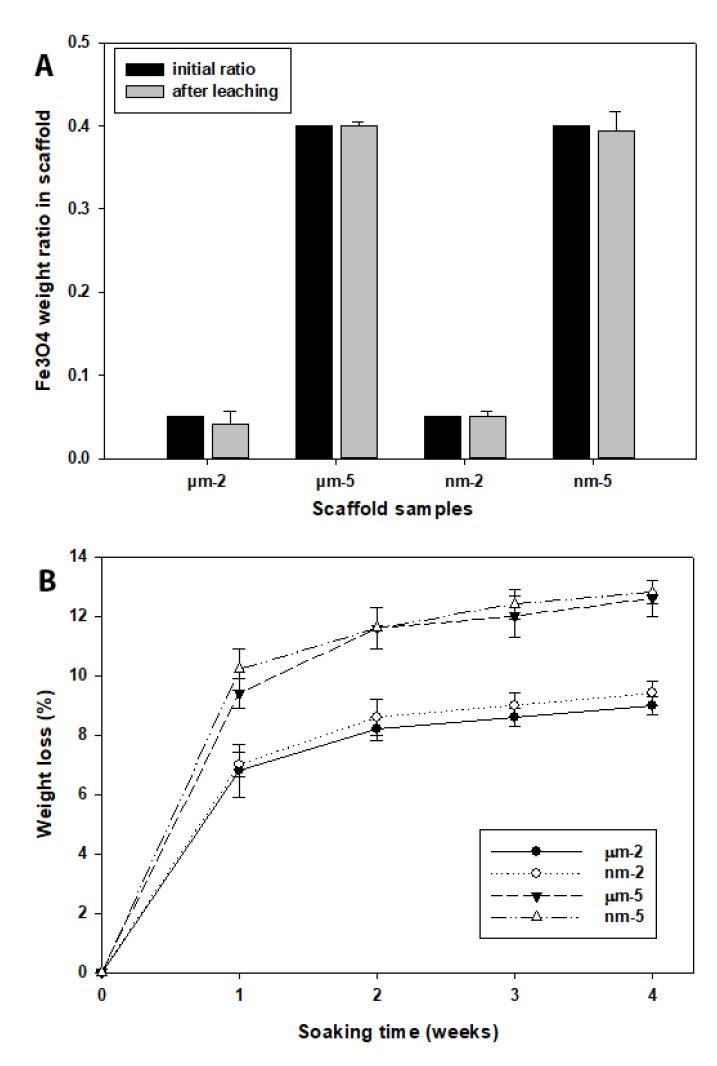
(**A**) Magnetic particle weight test in scaffolds of different groups. The final particle concentrations of the final scaffold products were compared with the initial weights included during the manufacture process. (**B**) Summary of the weight loss test of the testing scaffolds immersed in culture medium up to 4 weeks.

Figure 4A indicates the retention of the Fe_3_O_4_ particles in the scaffold during the fabrication process. After the removal of the organic polymers (PCL) of the final products of Fe_3_O_4_/PCL scaffolds, the actual metal particle weights were almost identical to the initial amount (Figure 4A). Further, an in vitro weight loss test was performed to examine the degradation rate of the scaffolds in a mimicked physiological condition. It appeared that the weight loss of the Fe_3_O_4_/PCL scaffolds was greater in samples containing more concentrated magnetic particles (40%) compared to the metal particles with a low concentration (5%). However, there was no degradation rate difference between the samples containing µm- or nm-sized particles throughout the test period (Figure 4B).

### 3.5. Evaluation of Cell Biocompatibility on the Fe_3_O_4_/PCL Scaffolds

**Figure 5:**
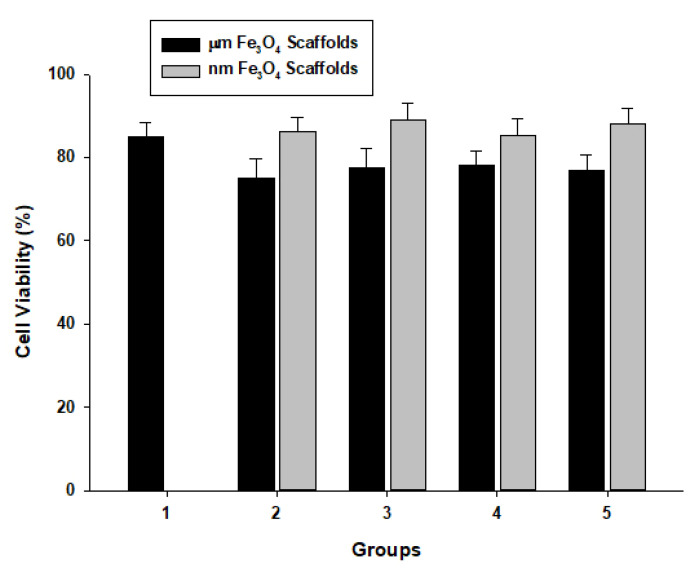
Cell biocompatibility of the magnetic scaffolds with various wt/wt ratios of Fe_3_O_4_ particles to PCL, i.e., 0:1 (1), 0.05:0.95 (2), 0.10:0.90 (3), 0.20:0.80 (4), and 0.40:0.60 (5).

MTT assay was used to evaluate the cell viability on the scaffolds. The data indicated that the addition of various sizes and amounts of Fe_3_O_4_ particles to the PCL scaffolds resulted in some influence on the cell viability as shown in Figure 5. There was higher cell viability in the samples with nm-sized Fe_3_O_4_ particles compared to the scaffolds with micron-sized Fe_3_O_4_ particles, although the difference was not significant (*p* = 0.22). It is worth mentioning that the high porosity and uneven surface areas of the samples may result in the penetration of cell growth within the scaffolds, and the actual cell viability patterns might be higher than what we found in Figure 5. Nevertheless, the cell viabilities of all samples were higher than 50%, suggesting that Fe_3_O_4_/PCL scaffolds are biocompatible and can be used in scaffolding.

### 3.6. Cytotoxicity Assay of the Fe_3_O_4_/PCL Scaffolds

Elution from different scaffolds of concentrated Fe_3_O_4_-PCL at different time points was added to 3T3 cell culture to determine the cytotoxicity of the magnetic scaffolds. Table 2 summarizes the cell viability after 2-day co-culturing. It appears that the cell viabilities in all tested medium samples from the Fe_3_O_4_/PCL scaffolds were higher than 70%, and there was no statistical difference between the treatments and the non-scaffold controls (*p* > 0.05). This means that the prepared Fe_3_O_4_/PCL scaffolds are nontoxic to the body cells.

## 4. Discussion

The current study investigated the in vitro characteristics of magnetic scaffolds for potential clinical applications, not only as a supporting scaffold, but also as a station for magnetic nanoparticle-drug delivery. The Fe_3_O_4_/PCL scaffolds prepared by the NaCl particulate leaching technique have open pores, and the pores connect with each other. Although the 355–500 µm-sized porogen (NaCl) was used, the final product resulted in a pore size range of 150–500 µm due to the pressure changes in the linear PCL polymers after leaching. However, this configuration of the scaffolds may help the growth of cells on them and maintain the desire stiffness [25]. At the same time, there are some small pores (5 μm to 100 μm) on the wall of large pores, which can help the transportation of small molecular nutrient substance in the scaffold and can consequently help the growth of cells on the scaffold.

Caution was paid to ensure the uniform distribution of magnetic particles within the scaffolds by actively stirring until mold casting. Examination of the leaching fluid confirmed that only a minimum trace of Fe particles was leached out. An additional experiment was performed to dissolve the PCL component of the final scaffolds and confirmed that the desired amount of magnetic particles was reserved. Based on previous studies in our laboratory on the amount of magnetic particles in the scaffolds [37,38], we evaluated four different concentrations of magnetic particles (5, 10, 20, 40% to PCL weight to weight ratio) in two particle size ranges (µm and nm) of the PCL scaffolds. The data showed that the magnetic property of the scaffolds was consistent among the samples in the same group, although it appeared that the magnetic performance in the micro-sized samples was different from that at the same concentration of magnetic scaffold samples with nano-sized particles, which may be related to the magnetic to superparamagnetic transition and magnetic domain ordinations.

Further, the data suggested that the present of Fe_3_O_4_ particles influenced the mechanical performances of the scaffolds in two ways. As Fe_3_O_4_ particles have higher stiffness than PCL, the presence of Fe_3_O_4_ particles increased the stiffness and Young’s modulus of the scaffolds. However, the magnetic particles may break the continuity of the polymer linkage that decreases the scaffold mechanical properties. In this study, the mechanical performance of the micron-sized sample, μm-3 (10% Fe_3_O_4_), appeared best compared to all other samples. On the other hand, all samples with nano-sized magnetic particles showed relative lower mechanical strength, probably due to their agglomeration, surface interactions, and lack of dispersion issues. Further investigations are warranted.

The degradation rate of a scaffold appears to be an important factor for bone regeneration and tissue engineering [39]. To determine the degradation rate of the magnetic particles containing scaffolds, a weight loss test was performed for four weeks. It appeared that the scaffolds with a higher ratio of Fe_3_O_4_ particles presented a faster degradation rate than the scaffolds with a lower concentration of Fe_3_O_4_ particles, with a weight loss of 12.8 ± 0.42% after four weeks of immersion in a normal culture medium.

Based on the cell biocompatibility data, the presence of the Fe_3_O_4_ particles influenced the cell biocompatibility of the scaffolds only at a very minimum. It is interesting that both the negative and the positive influences were mild, which means that the prepared Fe_3_O_4_/PCL scaffolds are biocompatible for use in scaffolding applications. We used a mouse fibroblastic cell line (3T3 cells) for the study since it is an immortalized cell line with a stable growth rate that has been widely used for biocompatibility investigation. The cell cytotoxicity assay of the scaffolds showed that all cell viabilities of the medium released from the Fe_3_O_4_/PCL scaffolds were higher than 70%. These data indicated that the Fe_3_O_4_/PCL scaffolds are nontoxic to the most common body cells and biocompatible overall.

Indeed, there are some limitations of this study. This in vitro investigation did not describe the long-term magnetic properties of the scaffolds and their safety issues, which will be included in subsequent in vivo investigations.

## 5. Conclusions

The PCL-based porous scaffolds were prepared using micron and nanoscale Fe_3_O_4_ particles. The data suggested that the Fe_3_O_4_/PCL scaffolds prepared by the particulate leaching technique had a good configuration and a high porosity percentage that can help the growth of cells. The Fe_3_O_4_ particles had a greater influence on the mechanical performances of the prepared scaffolds when they were at a micron scale (Young’s modulus was high, reaching 1 MPa, and the stiffness was high, reaching 13 N/mm), which may be related to dispersion, agglomeration, and bonding issues. The presence of the Fe_3_O_4_ particles influenced the cell biocompatibility of the scaffolds to different degrees, but both the negative and positive influences were mild. The cytotoxicity study shows that the prepared Fe_3_O_4_/PCL scaffolds are nontoxic. Animal experiments are being conducted to evaluate the usefulness of these scaffolds for tissue engineering applications.

## Figures and Tables

**Table 1:** Preparations of Fe_3_O_4_/PCL scaffolds with different ratios of inclusions.

Sample	Fe_3_O_4_ Size	Fe_3_O_4_ (*w*/*w*, %)	PCL (*w*/*w*, %)
1	-	0	100
μm-2	<5 μm	5	95
μm-3	<5 μm	10	90
μm-4	<5 μm	20	80
μm-5	<5 μm	40	60
nm-2	<50 nm	5	95
nm-3	<50 nm	10	90
nm-4	<50 nm	20	80
nm-5	<50 nm	40	60

**Table 2:** Cytotoxicity assay of the prepared magnetic scaffolds as a function of time (day 1–day 7). Tests included micro- and nano-meter sized Fe_3_O_4_ at different ratios to PCL.

**Days**		**1**	**2**	**3**	**4**	**5**	**6**	**7**
**Fe_3_O_4_ free**		0.98 ± 0.04	0.97 ± 0.02	0.98 ± 0.01	0.88 ± 0.09	1.10 ± 0.04	1.09 ± 0.05	1.04 ± 0.02
µm Fe_3_O_4_ Scaffold	5%	0.92 ± 0.03	0.95 ± 0.05	0.90 ± 0.04	0.82 ± 0.05	0.94 ± 0.05	0.99 ± 0.10	0.94 ± 0.05
	10%	0.94 ± 0.04	0.94 ± 0.03	0.95 ± 0.02	0.88 ± 0.03	1.01 ± 0.02	0.97 ± 0.04	0.95 ± 0.04
	20%	1.03 ± 0.04	0.98 ± 0.05	0.90 ± 0.05	0.81 ± 0.04	0.99 ± 0.05	0.97 ± 0.05	0.95 ± 0.05
	40%	0.97 ± 0.05	0.90 ± 0.04	0.89 ± 0.05	0.80 ± 0.08	0.94 ± 0.04	0.94 ± 0.06	0.95 ± 0.05
nm Fe_3_O_4_ Scaffold	5%	1.01 ± 0.07	0.96 ± 0.05	0.94 ± 0.05	0.81 ± 0.05	1.05 ± 0.04	1.08 ± 0.04	1.02 ± 0.02
	10%	0.99 ± 0.05	0.97 ± 0.06	0.90 ± 0.06	0.79 ± 0.06	0.97 ± 0.05	0.98 ± 0.05	1.02 ± 0.05
	20%	0.92 ± 0.03	0.88 ± 0.05	0.87 ± 0.05	0.79 ± 0.05	0.96 ± 0.08	0.97 ± 0.04	1.00 ± 0.03
	40%	0.92 ± 0.05	0.89 ± 0.01	0.85 ± 0.03	0.81 ± 0.03	0.95 ± 0.05	0.97 ± 0.05	0.95 ± 0.06

## Data Availability

The original data/images can be obtained by requesting to the first and corresponding authors.
